# Arenaviral vaccine vectors to combat infectious diseases

**DOI:** 10.18632/oncotarget.10405

**Published:** 2016-07-06

**Authors:** Rekha Dhanwani, Hinh Ly

**Affiliations:** Department of Veterinary & Biomedical Sciences, University of Minnesota, Twin Cities, MN, USA

**Keywords:** arenavirus, vaccine vector, LCMV, pichinde virus, reverse genetics

In recent years, the use of viral vaccine vectors has gained immense interest over traditional vaccine strategies due to their ability to replicate in order to induce high immune responses against the intended antigens without the need for adjuvant co-administration. Several viral vectors, including but not necessarily limited to adenoviruses, poxviruses and alphaviruses are currently in various preclinical and clinical stages of development [1]. However, due to the inherent risk of pathogenesis associated with these live viral vectors, there is continuing effort to search for other viral vectors that can offer a good safety profile without compromising immunogenicity. The recent development of recombinant arenaviruses as new vaccine vectors has offered some additional advantages over other existing viral vectors.

Arenaviruses are enveloped bi-segmented negative strand RNA viruses that encode four genes. The L segment encodes for the RNA-dependent RNA polymerase L and the ring-finger motif containing Z protein. The S segment encodes for the nucleoprotein NP and the glycoprotein GPC. These viruses target the antigen-presenting cells (i.e., macrophages and dendritic cells) early in the infection and have developed different strategies to evade host innate immune recognition [2].

The recent development of reverse genetics systems to generate recombinant arenaviruses has allowed for convenient and effective expression of foreign antigens directly from the viral genome. The use of arenaviruses as vaccine vectors was first reported in 2009 by Emonet and colleagues, who attempted to express the eGFP and CAT reporter genes as foreign antigens from the engineered tri-segmented genome of the lymphocytic chroriomengingitis virus (LCMV) [3]. By doing so, they showed that the expression levels of these foreign genes of interest were comparable to the expression levels of indigenous viral gene products. However, this study did not use any bona fide immunogenic antigens in order to evaluate the efficacy of the vaccine vector. In order to further evaluate this concept, we have recently developed a tri-segmented recombinant Pichinde virus (triPICV) as a new vaccine vector [4]. Unlike LCMV, which has the potential to cause diseases in humans and a relatively high seroprevalence in human population, PICV is generally not known to be pathogenic in humans, who would rarely come in contact with this virus due to the fact that it is only found in Colombia, South America. Therefore, this viral vector can overcome a major challenge of preexisting vector immunity that often limits the use of viral vectors as vaccines.

We have used the recombinant triPICV to deliver different foreign antigens, including the influenza hemagglutinin (HA) and nucleoprotein (NP) that could provide complete protection against lethal influenza virus challenge in a mouse model [4]. Specifically, this viral vector could mount robust influenza antigen-specific humoral and cellular immune responses and proved ideal for use in a prime-boost vaccination strategy. An added advantage of the recombinant triPICV is that it induces very low anti-PICV immunity in vaccinated animals, due possibly to the fact that arenaviral envelop glycoproteins are highly glycosylated, which appear to play an important role in dampening the development of neutralization antibodies against these viruses [5].

Due to the aforementioned reasons, arenaviruses have emerged as a promising vaccine vector platform. The ability to incorporate multiple foreign antigens into their genome and their ability to mount robust humoral and cell-mediated immune responses in the vaccinated hosts even after repeated administrations are some of the unique features of these viral vectors. In addition, it appears that arenaviruses (e.g., PICV) have a relatively wide tropism against many different cell types and organisms. Therefore, the development of arenavirus-based vaccine vectors adds to the existing arsenal of viral vaccine vectors for consideration of testing in preclinical and clinical trials against infectious diseases.
